# Supplementary material for: Exploring the Impact of Terminators on Transgene Expression in Chlamydomonas reinhardtii with a Synthetic Biology Approach
Source: Life (Basel). 2021 Sep 14;11(9):964. doi: 10.3390/life11090964 (PMC8471596; doi:10.3390/life11090964)
Supplement: Supplementary file 1 [file life-11-00964-s001.zip › Supplementary material - Geisler et al revised.pdf]

life

MDPI

Supplementary Materials

Exploring the impact of terminators on transgene expression in *Chlamydomonas reinhardtii* with a synthetic biology approach.

Katrin Geisler [1], Mark A. Scaife [1,2], Paweł M. Mordaka [1], Andre Holzer [1], Eleanor V. Tomsett [1], Payam Mehrshahi [1], Gonzalo I. Mendoza Ochoa [1] and Alison G. Smith [1,*]

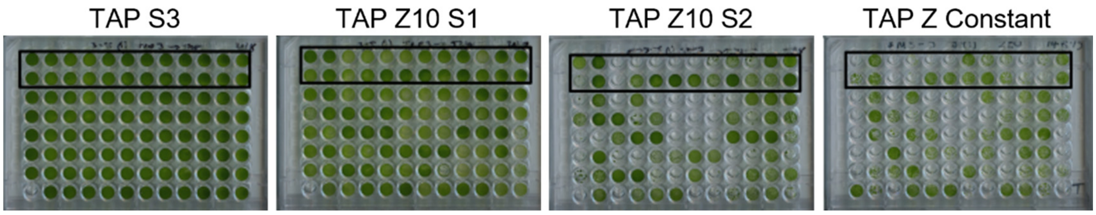

Figure S1. Comparison of no selection versus selection workflow. Example images depict growth in TAP after 3 subcultures (TAP S3), the subsequent transfer from TAP S3 into TAP containing zeocin (TAP Z10 S1), the second subculture in the presence of zeocin (TAP Z10 S2) and for comparison the replica plates that have been cultured continuously in the presence of zeocin (TAP Z10 constant). All images were taken on day 7 of growth. Black boxes show cell lines analyzed further for GFP expression.

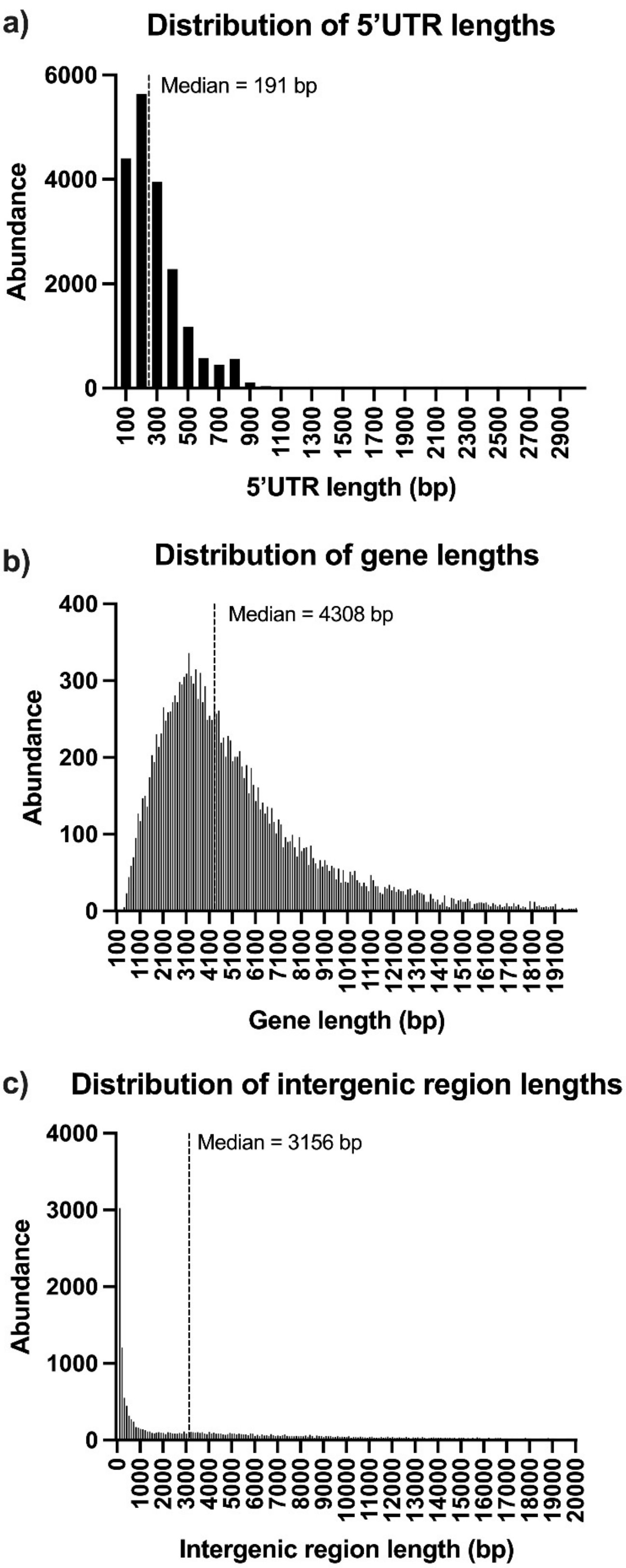

Figure S2. Analysis of genomic features in *C. reinhardtii*. (a) Distribution of 5′ UTR lengths. (b) Distribution of gene lengths. (c) Distribution of the length between intergenic regions. All lengths are based on annotations from the *C. reinhardtii* genome v5.6. Calculated median sizes of genomic features are shown.

Table S1. Primers used for Gibson assembly and DNA part generation.

Part Name	Primer Name	Primer Sequence (5′-3′)
GFP-3′UTR	Rbcs2(B)-Ble F	GAGAAGTCACTCAACATCTTAAATGGCCAAGCTGACCAGCGCCGTTC
GFP-RPL31	GFP-L31 R	CCACAGAAAGAGTTACACACTTGCGGCGTATGGCTTACTTGTACAGCTCGTCCATGCCGT
GFP-RPS29	GFP-RPS29 R	ACGGCATGGACGAGCTGTACAAGTAAAATGCCCGAATGTTGGGTATCTAGCTCACACG CAGTTTGTAAGGTGCTGAGGCGTTG
GFP-RPL11	GFP-RPL11 R	GGTCAGGACTTGTTACACAGATGGGGTGGCATCTGTCATCACCAGCCTCTTGTGGCCGCT TTACTTGTACAGCTCGTCCATGCCGT
GFP-RBCS2	GFP-RBCS2 R	GCTCAGATCAACGAGCGCCTCCATTTACTTGTACAGCTCGTCCATGCCGT
GFP-PSAD	GFP-PSAD R	GGTACAGGCGGTCCAGCTGCTGCCTTACTTGTACAGCTCGTCCATGCCGT
GFP-THI4	GFP-THI4 R	GCAGCCCAGTAGGTTCTGAGCGCGCTTACTTGTACAGCTCGTCCATGCCGT
GFP-METE	GFP-METE R	TCAGCATAAATCAAGGCAGGCAGCTTACTTGTACAGCTCGTCCATGCCGT
GFP-CA1	GFP-CA1 R	TAGCGTGACTAACTACTGGGAAGTTTACTTGTACAGCTCGTCCATGCCGT
GFP-NIT1	GFP-NIT1 R	ACCGTGGCCACAATCTCTAGCGATTTACTTGTACAGCTCGTCCATGCCGT
GFP-noUTR	GFP-noUTR R	GTGCGTCGGGTGATGCTGCCAACTTACTGATTTAGTTACTTGTACAGCTCGTCCATGCCGT
noUTR	GFP-noUTR F	ACGGCATGGACGAGCTGTACAAGTAACTAAATCAGTAAGTTGGCAG
RBCS2	GFP-RBCS2 F	ACGGCATGGACGAGCTGTACAAGTAAATGGAGGCGCTCGTTGATCTGAGC
RBCS2	RBCS2-BADCmR	GTGATGCTGCCAACTTACTGATTTAGCGCTTCAAATACGCCCAGCCCGCC
PSAD	GFP-PSAD F	ACGGCATGGACGAGCTGTACAAGTAAGGCAGCAGCTGGACCGCCTGTACC
PSAD	PSAD-BADCmR	GTGATGCTGCCAACTTACTGATTTAGCACGGCAAACTCTCACATGGCCTG
THI4	GFP-THI4 F	ACGGCATGGACGAGCTGTACAAGTAAGCGCGCTCAGAACCTACTGGGCTGC
THI4	THI4-BADCmR	GTGATGCTGCCAACTTACTGATTTAGTCTCAACTCCAAATTGTTACATGA
METE	GFP-METE F	ACGGCATGGACGAGCTGTACAAGTAAGCTGCCTGCCTTGATTTATGCTGA
METE	METE-BADCmR	GTGATGCTGCCAACTTACTGATTTAGACGTCACACCTTGCTACCCTACAG
CA1	GFP-CA1 F	ACGGCATGGACGAGCTGTACAAGTAAACTTCCCAGTAGTTAGTCACGCTA
CA1	CA1-BADCmR	GTGATGCTGCCAACTTACTGATTTAGTCCATGGGCATCTTACATGTCGTA
NIT1	GFP-NIT1 F	ACGGCATGGACGAGCTGTACAAGTAAATCGCTAGAGATTGTGGCCACGGT
NIT1	NIT1-BADCmR	GTGATGCTGCCAACTTACTGATTTAGCGCTTGCTTAATTTACAACTGTGC
RPL31-pUC	L31-BADCmF	GCAAGTGTGTAACTCTTTCTGTGGCTAAATCAGTAAGTTGGCAGCATCAC
RPS29-pUC	RPS29-BADCmF	AATGCCCGAATGTTGGGTATCTAGCTCACACGGCAGTTTGTAAGGTGCTGAGGCGTT-GCTAAATCAGTAAGTTGGCAGCATCAC
RPL11-pUC	RPL11-BADCmF	AGCGGCCACAAGAGGCTGGTGATGACAGATGCCACCCCATCTGTG-TAACAAGTCCTGACCCTAAATCAGTAAGTTGGCAGCATCAC
RBCS2-pUC		GGCGGGCTGGGCGTATTTGAAGCGCTAAATCAGTAAGTTGGCAGCATCAC
PSAD-pUC	PSAD-BADCmF	CAGGCCATGTGAGAGTTTGCCGTGCTAAATCAGTAAGTTGGCAGCATCAC
THI4-pUC	THI4-BADCmF	TCATGTAACAATTTGGAGTTGAGACTAAATCAGTAAGTTGGCAGCATCAC
METE-pUC	METE-BADCmF	CTGTAGGGTAGCAAGGTGTGACGTCTAAATCAGTAAGTTGGCAGCATCAC
CA1-pUC	CA1-BADCmF	TACGACATGTAAGATGCCCATGGACTAAATCAGTAAGTTGGCAGCATCAC
NIT1-pUC	NIT1-BADCmF	GCACAGTTGTAAATTAAGCAAGCGCTAAATCAGTAAGTTGGCAGCATCAC
pUC-AR	pUC-AR R	CACCAATCATGTCAAGCCTCAGCGAGCTCCCCGCCGTCGTACCGAGCTCGAATTCG-TAATCATGGTCA
pUC-PSAD	pUC-PSAD R	TGACCATGATTACGAATTCGAGCTCGGTACATCCCACACACCTGCCCGTCTGCCTGACA
pUC-METE	pUC-METE R	GATTACGAATTCGAGCTCGGTACGTAGGTCAGGACCAGAGCCTACAAC
AR prom	pUC-AR F	CGAATTCGAGCTCGGTACGACGGCGGGGAGCTCGCTGAGGCTTGACATGATTGGTGCG-TATGTTTG
AR prom	AR-Ble R	GAACGGCGCTGGTCAGCTTGGCCATTTAAGATGTTGAGTGAC
PSAD prom	pUC-PSAD F	TGACCATGATTACGAATTCGAGCTCGGTACATCCCACACACCTGCCCGTCTGCCTGACA
PSAD prom	pPSAD-Ble R	ACTGCTACTCACAACAAGCCCATGGCCAAGCTGACCAGCGCCGTT
METE prom	pUC-METE R	TGACCATGATTACGAATTCGAGCTCGGTACGTAGGTCAGGACCAGAGCCTACAAC
METE prom	pMETE-Ble R	CAGCTTGGCCATTTTAAGATGTTGAGTGACATGTCACTTAAATAATCGGCCTG
KG0-140	BleGFP.Fw	TTGAAGACATAATGGCCAAGCTGACCAGC
KG0-140	BleGFP.Rv	TTGAAGACATCGAACCCTTGTACAGCTCGTCCATG

pKG0-472	THI43U.Fw.gg	TTGAAGACATGCTTGCGCGCTCAGAACC
pKG0-472	THI43U.Rv.gg	TTGAAGACATAGCGTCTCAACTCCAAATTGTTACATGAG
pKG0-576	NIT13U.Fw.gg	TTGAAGACATGCTTATCGCTAGAGATTGTGGC
pKG0-576	NIT13U.Rw.gg	TTGAAGACATAGCGCGCTTGCTTAATTTACAACTG
pKG0-577	METE3U.Fw.gg	TTGAAGACATGCTTGCTGCCTGCCTTGATTTATG
pKG0-577	METE3U.mBpi.gg	TTGAAGACATTgTTCTGTCGGATGAGTTCAAGG
pKG0-577	METE3UmBpiFw2	TTGAAGACATAAcACCGTATATGAGCTGGTGGCCTGTAGGGTAGCAAGGTGTGACGT
pKG0-577	METE3UmBpiRv2	TTGAAGACATAGCGACGTCACACCTTGCTACCCTACAGGCCACCAGCTCATATACGGT

Table S2. List of plasmids generated by Gibson assembly.

Plasmid	Terminator tested	Description	Figure
pMS3-0	-	*pAR::Ble-GFP*	3, 4, 5
pMS3-8	*RPL31*	*pAR::Ble-GFP::tRPL31*	3
pMS3-1	*RPS29*	*pAR::Ble-GFP::tRPL29*	3, 4
pMS3-3	*RPL11*	*pAR::Ble-GFP::tRPL11*	3
pMS3-14	*RBCS2*	*pAR::Ble-GFP::tRBCS2*	3
pMS3-6	*PSAD*	*pAR::Ble-GFP::tPSAD*	3, 4, 5
pMS3-11	*THI4*	*pAR::Ble-GFP::tTHI4*	3
pMS3-10	*METE*	*pAR::Ble-GFP::tMETE*	3
pMS3-12	*CA1*	*pAR::Ble-GFP::tCA1*	3, 4, 5
pMS3-13	*NIT1*	*pAR::Ble-GFP::tNIT1*	3
pMS3-N	*PSAD*	*pPSAD::Ble-GFP::tPSAD*	5
pMS3-K	*CA1*	*pPSAD::Ble-GFP::tPSAD*	5
pMS3-O	*PSAD*	*pMETE::Ble-GFP::tPSAD*	5
pMS3-L	*CA1*	*pMETE::Ble-GFP::tPSAD*	5

Table S3. MoClo constructs employed and generated. The level 2 plasmids were used to generate data for Figure 6.

Plasmid	Description	Function	Level	Source
pCM0-010	*pPSAD (Pro + 5′UTR)*	Promoter + 5′UTR	0	[1]
pCM0-011	*pAR (Pro + 5′UTR)*	Promoter + 5′UTR	0	[1]
pPM0-024	*RBCS2i1 (5′UTR)*	RBCS2 intron 1 as 5′UTR enhancer	0	This study
pKG0-140	*BleGFP (CDS)*	Resistance to zeocin + Reporter GFP	0	This study
pCM0-098	*HA (C-ter tag, CDS)*	Immuno- and purification tag	0	[1]
pCM0-074	*AphVIII (CDS)*	Resistance to paromomycin	0	[1]
pCM0-116	*RSP29 (3′UTR + Ter)*	3'UTR and Terminator	0	This study, [1]
pCM0-114	*PSAD (3′UTR + Ter)*	3'UTR and Terminator	0	[1]
pCM0-117	*CA1 (3′UTR + Ter)*	3'UTR and Terminator	0	This study, [1]
pKG0-472	*THI4 (3′UTR + Ter)*	3'UTR and Terminator	0	This study
pKG0-576	*NIT1 (3′UTR + Ter)*	3'UTR and Terminator	0	This study
pKG0-577	*METE (3′UTR + Ter)*	3'UTR and Terminator	0	This study
pFL_L1_004	*pPSAD::RBCS2i1::BleGFPHA::tRPS29*	Expression cassette	1	This study
pFL_L1_005	*pPSAD::RBCS2i1::BleGFPHA::tPSAD*	Expression cassette	1	This study
pFL_L1_006	*pPSAD::RBCS2i1::BleGFPHA::tCA1*	Expression cassette	1	This study
pFL_L1_011	*pAR::RBCS2i1::AphVIII::tPSAD*	Expression cassette	1	This study
pFL_L2_038	*pPSAD::RBCS2i1::BleGFPHA::tRPS29::: pAR::RBCS2i1::AphVIII::tPSAD*	Expression cassette	2	This study
pFL_L2_044	*pPSAD::RBCS2i1::BleGFPHA::tPSAD::: pAR::RBCS2i1::AphVIII::tPSAD*	Expression cassette	2	This study
pFL_L2_050	*pPSAD::RBCS2i1::BleGFPHA::tCA1:::*	Expression cassette	2	This study

	pAR::RBCS2i1::AphVIII::tPSAD

Table S4. Expression ranking of *C. reinhartii* genes used in this study. The expression rank over the diurnal cycle was determined by comparing the mean FPKM values in the dark, in the light and over the diurnal cycle. Data obtained from Strenkert *et al.* [2] (Dataset S2). The mean FPKM values (Mean), standard deviation (SD) and the expression rankings (Rank) are listed.

Gene ID	Name	Expression in the dark[1]			Expression in the light[2]			Expression diurnal cycle[3]		
		Mean	SD	Rank	Mean	SD	Rank	Mean	SD	Rank
Cre12.g489153	*L31*	2023.91	176.15	40	2571.08	903.89	45	2425.76	803.35	42
Cre08.g358556	*RPS29*	1312.28	125.52	82	1663.83	599.01	84	1597.48	563.75	79
Cre01.g027000	*RPL11*	1578.22	131.49	61	1946.29	693.82	68	1889.14	651.09	63
Cre02.g120150	*RBCS2*	5281.06	713.44	13	8078.13	2585.11	15	7054.93	2384.99	12
Cre05.g238332	*PSAD*	974.70	349.65	116	2425.35	1012.79	51	1577.50	942.19	80
Cre04.g214150	*THI4*	463.50	157.47	188	736.43	520.54	179	522.81	375.75	194
Cre03.g180750	*METE*	39.82	90.98	812	396.42	176.57	232	173.57	214.41	345
Cre04.g223100	*CA1*	42.56	44.69	771	417.34	343.95	225	217.22	268.27	300
Cre09.g410950	*NIT1*	0.14	0.14	14971	0.13	0.04	15373	0.13	0.05	15537

[1] For expression in the dark, FPKM values from timepoints -11,-9,-7,-5,-3,-1h were used.

[2] For expression in the light, FPKM values from timepoints 1,3,5,7,9,11h were used.

[3] For expression over the diurnal cycle FPKM values from all timepoints were used.

References

1. Crozet, P.; Navarro, F.J.; Willmund, F.; Mehrshahi, P.; Bakowski, K.; Lauersen, K.J.; Pérez-Pérez, M.-E.; Auroy, P.; Gorchs Rovira, A.; Sauret-Gueto, S.; et al. Birth of a Photosynthetic Chassis: A MoClo Toolkit Enabling Synthetic Biology in the Microalga Chlamydomonas reinhardtii. *ACS Synth. Biol.* **2018**, *7*, 2074–2086, doi:10.1021/acssynbio.8b00251.
2. Strenkert, D.; Schmollinger, S.; Gallaher, S.D.; Salomé, P.A.; Purvine, S.O.; Nicora, C.D.; Mettler-Altmann, T.; Soubeyrand, E.; Weber, A.P.M.; Lipton, M.S.; et al. Multiomics resolution of molecular events during a day in the life of Chlamydomonas. *Proc. Natl. Acad. Sci. U. S. A.* **2019**, *116*, 2374–2383, doi:10.1073/pnas.1815238116.
